# The Role of Alpha-Synuclein in Melanin Synthesis in Melanoma and Dopaminergic Neuronal Cells

**DOI:** 10.1371/journal.pone.0045183

**Published:** 2012-09-19

**Authors:** Tianhong Pan, Julie Zhu, Wen-Jen Hwu, Joseph Jankovic

**Affiliations:** 1 Neurology Department, Parkinson Disease Research Laboratory, Baylor College of Medicine, Houston, Texas, United States of America; 2 Melanoma Medical Oncology, M.D. Anderson Cancer Center, Houston, Texas, United States of America; 3 Parkinson’s Disease Center and Movement Disorder Clinic, Baylor College of Medicine, Houston, Texas, United States of America; McGill University Department of Neurology and Neurosurgery, Canada

## Abstract

The relatively high co-occurrence of Parkinson’s disease (PD) and melanoma has been established by a large number of epidemiological studies. However, a clear biological explanation for this finding is still lacking. Ultra-violet radiation (UVR)-induced skin melanin synthesis is a defense mechanism against UVR-induced damage relevant to the initiation of melanoma, whereas, increased neuromelanin (NM), the melanin synthesized in dopaminergic neurons, may enhance the susceptibility to oxidative stress-induced neuronal injury relevant to PD. *SNCA* is a PD-causing gene coding for alpha-Synuclein (α-Syn) that expresses not only in brain, but also in skin as well as in tumors, such as melanoma. The findings that α-Syn can interact with tyrosinase (TYR) and inhibit tyrosine hydroxylase (TH), both of which are enzymes involved in the biosynthesis of melanin and dopamine (DA), led us to propose that α-Syn may participate in the regulation of melanin synthesis. In this study, by applying ultraviolet B (UVB) light, a physiologically relevant stimulus of melanogenesis, we detected melanin synthesis in A375 and SK-MEL-28 melanoma cells and in SH-SY5Y and PC12 dopaminergic neuronal cells and determined effects of α-Syn on melanin synthesis. Our results showed that UVB light exposure increased melanin synthesis in all 4 cell lines. However, we found that α-Syn expression reduced UVB light-induced increase of melanin synthesis and that melanin content was lower when melanoma cells were expressed with α-Syn, indicating that α-Syn may have inhibitory effects on melanin synthesis in melanoma cells. Different from melanoma cells, the melanin content was higher in α-Syn-over-expressed dopaminergic neuronal SH-SY5Y and PC12 cells, cellular models of PD, than that in non-α-Syn-expressed control cells. We concluded that α-Syn could be one of the points responsible for the positive association between PD and melanoma via its differential roles in melanin synthesis in melanoma cells and in dopaminergic neuronal cells.

## Introduction

Melanoma is the most dangerous form of skin cancers, characterized by uncontrolled growth of skin melanocytes, whereas, Parkinson’s disease (PD) is one of the neurodegenerative disorders, characterized by a progressive loss of pigmented dopamine (DA) neurons in the substantia nigra (SN). Although PD and melanoma are two different diseases, numerous epidemiological studies have established an increased incidence of melanoma in patients with PD and vice verse [1]–[5]. However, the pathogenic pathways responsible for this link have not yet been fully defined [6]–[10].

Exposure of skin to sunlight or to tanning machines and in particular exposure to ultra-violet radiation (UVR) may lead to DNA damage-initiated development of melanoma [11]–[13], which process can be prevented by skin melanin. Thus, skin melanin synthesis is considered as a defense mechanism against UVR-induced initiation of melanoma [14], [15]. Melanin is a determinant of skin color. Light skin color such as that in Caucasian populations contains less melanin. The findings that the incidence of both melanoma [14], [16], [17] and PD [18]–[20] is higher in Caucasian populations than that in black populations indicate that in addition to melanoma, low skin melanin level may also enhance the vulnerability to PD.

Although the etiology of dopaminergic neuronal degeneration in PD remains unknown, alpha-Synuclein (α-Syn) gene (*SNCA*) has been implicated in the pathogenesis of both familial and sporadic PD [21]–[23]. α-Syn, predominantly expressed in the brain, has been found to accumulate in the peripheral nervous system [24]–[26] and to be directly transmitted from pathologically affected neurons to healthy, unaffected cells [27], [28]. α-Syn is also reported to be expressed in skin [26] as well as in various tumors including ovarian and breast [29], colorectal [30] and melanoma [31]. Tyrosinase (TYR) and tyrosine hydroxylase (TH) are enzymes involved in the biosynthesis of melanin and dopamine (DA) initiated by the conversion of tyrosine to DOPA [32]–[34]. The findings that α-Syn can interact with TYR [35] and inhibit TH [32], raise the possibility that α-Syn may play a role in regulating the biosynthesis of melanin and DA.

Ultra-violet B (UVB) light is physiologically relevant to UVR that may stimulate melanogenesis by enhancing melanin synthesis via an enzymatic cascade controlled by certain pigmentation genes [37], [38]. In this study, we applied UVB light on SK-MEL-28 and A375 human melanoma cells to determine roles of α-Syn in melanin synthesis. We also used α-Syn over-expressed SH-SY5Y cells and PC12 as PD cellular models to determine roles of α-Syn in melanin synthesis in dopaminergic neuronal cells. We found that α-Syn plays differential roles in melanin synthesis in melanoma and dopaminergic neuronal cells, which may explain the increased incidence of melanoma in PD patients.

## Materials and Methods

### Cell Cultures and Transfection

A375 melanoma cells (ATCC, Manassas, VA) and SH-SY5Y cells [39], [40] were routinely grown in Dulbecco’s modified Eagle’s medium (DMEM) supplemented with 10% heat-inactivated fetal bovine serum (FBS) (GIBCO, Gaithersburg, MD). SK-MEL-28 melanoma cells (ATCC, Manassas, VA) known to have high expression of α-Syn [31] were maintained in Eagle’s Minimum Essential medium (EMEM) (ATCC, Manassas, VA) supplemented with 10% FBS. Stable inducible PC12 cells expressing HA-tagged wild-type α-Syn (kind gift of professor David Rubinsztein from Department of Medical Genetics, Cambridge Institute for Medical Research, University of Cambridge) [41], [42] were maintained at 70 µg/ml hygromycin B (Calbiochem), 50 µg/ml G418 (Sigma), 10% horse serum and 5% FBS DMEM. All cells were cultured at 37°C under humidified 5% CO_2_ atmosphere.

To silence endogenous α-Syn expression, SK-MEL-28 melanoma cells were transfected with small interference RNA (siRNA) of *SNCA* (siRNA ID S13206, Ambion INC, Austin, TX, USA) at concentration of 100 nM using lipofectamine™ 2000 (Invitrogen, Carlsbad, CA, USA) as we described previously [43]. Silencer™ negative control #3 siRNA (Ambio INC), with no significant homology to any known gene sequences from mouse, rat and human, was used as a sham control. To establish cells with wild type (wt)-α-Syn over-expression, A375 melanoma cells and SH-SY5Y cells were transfected with wt-α-Syn expression plasmid (Kind gift from Dr. Chuantao Jiang, Research Center for Protein Chemistry, Department of Biochemistry and Molecular Biology, The University of Texas, Houston, Texas) [44] using lipofectamine™ 2000. Cells transfected with pGEX-1 control vector were used as control. In order to maintain consistent levels of insert α-Syn expression between experiments, 48 h after transfection, A375 and SH-SY5Y cells were exposed to medium containing G418 (800 µg/ml) for 2 weeks. The selected polyclonal pools of stable transfected cells were passed and maintained in culture medium containing G418 (200 µg/ml) for experimental assays. The expression of wt-α-Syn in stable inducible PC12 cells was induced by 1 µg/ml doxycycline (Sigma) for 48 h as we described previously [45].

### UVB Light Irradiation

Irradiation was conducted using a 6W-power UVB lamp (Cole-Parmer, Vernon Hills, IL, USA) with a wavelength spectrum of 280–320 nm. Sub-confluent cells were washed and irradiated in phosphate buffered saline (PBS) with energy of UVB emission amounted to 2.2 mW/cm^2^ at a target distance of 3 inches. The exposure of cells to UVB light for 13, 54, and 110 seconds corresponds to doses of 30, 120, and 240 mJ/cm^2^ UVB respectively. Immediately after irradiation, the cells were changed with fresh cell culture medium and returned to the incubator and cultivated for the indicated time periods [46], [47].

### Cell Viability

The general viability of cultured cells were determined by reduction of MTT (3-(4,5-dimethylthiazol-2-yl)-2,5-diphenyltetrazoliumbromide; Sigma, St. Louis, MI) to formazan as we reported previously [48]. The cytotoxicity-based MTT assay was performed with three UVB radiation doses: 30, 120 and 240 mJ/cm^2^ and post-cultured for 4, 24 and 48 h.

### Total Melanin Content

The melanin content was determined according to previous publications with modifications [37], [49]–[51]. Briefly, the content of extracellular melanin was measured directly from cell culture medium spectrophotometrically at 415 nm using iMark Microplate Reader. For the cellular melanin, the adherent cells were washed with PBS and detached by trypsinization, resuspended in culture medium. Fifty microliters of cell suspension were used for counting cell numbers with hemacytometer under microscope. Other cell suspensions were centrifuged and re-suspended in 1 M NaOH and incubated at 80°C for 30 minutes followed by centrifugation at 1,500 rpm for 5 minutes. The absorbance of the supernatant was measured at 415 nm. The amount of melanin was compared with the standard curve of the serial dilution of standard melanin (Sigma, USA) and was normalized to the total number of cells and expressed as microgram per 1×10^4^ cells (µg/10^4^ cells). The total amount of melanin was come from the sum of extracellular (medium) and intracellular melanin.

### Tyrosinase (TYR) Enzyme Activity

TYR enzyme activity was estimated by measuring the rate of L-DOPA (3,4-dihydroxyphenylalanine) oxidation as previously described [37], [50] with slight modifications. Briefly, after specific treatment, the adherent cells were washed three times with PBS and then lysed in PBS containing 1% Triton X-100. After protein quantification, equal amount of protein were aliquoted into the wells of a 96-well plate. Then, 20 µl of 5 mM L-DOPA was added to each plate well, incubated at 37°C and allowed to react for 45 min. The end-point absorbance was measured spectrophotometrically at 475 nm using iMark Microplate Reader. The results were expressed as percentage of non-UVB control.

### Release of Dopamine (DA) as Determined by Extra-cellular DA Content

The release of DA was determined by measuring DA content in cell culture medium using high-pressure liquid chromatography (HPLC) according to the method we described previously [48]. Briefly, cell culture medium 100 µl was lysed with 50 µl of 0.1 M perchloride acid. Homogenates were centrifuged at 13,000 rpm for 10 min at 4°C. The resulted supernatants were filtered by acro-disc filters (mesh size, 0.25 µm Fisher, Scientific, Houston, TX) before being subjected to HPLC assay. The DA content was normalized to the total amount of protein and expressed as nanogramme (ng) per milligram (mg) protein (ng/mg protein) and results were expressed as percentage of non-UVB control.

### Protein Isolation and Western Blot Analysis

Total proteins were isolated with mammalian tissue lysis/extraction reagent supplemented with protease inhibitor cocktails (Sigma, St. Louis, MO). The equal amounts of protein were subjected to western blot assay as we described previously [39], [45]. The protein levels of α-Syn and TYR were determined using anti-α-Syn [4D6] antibody (Abcam) and anti-TYR antibody (Santa Cruz Biotechnology, Santa Cruz, CA). Immunoblot of *β*-actin (Santa Cruz Biotechnology, Inc) was performed to demonstrate equal protein loading.

### RNA Isolation and Real-time Quantitative PCR

Total RNA was extracted from cells with Direc-zol™ RNA Miniprep kit (The Epigenetics Company). The iScript one-step RT-PCR kit with SYBR Green (Bio-rad) was used for real-time quantitative PCR of RNA templates, in which, cDNA synthesis and PCR amplification were carried out in the same tube. Specific primers for human TYR were based on published nucleotide sequences (forward: 5′-GGCTGTTTTGTACTGCCTGCT-3′; reverse: 5′ AGGAGACACAGGCTCTAGGGAA-3′) [53]. Human *ß*-actin gene was used as internal control. Specific primers for *ß*-actin were designed using primer 3 and BLAST system (NCBI) (forward: 5′-GGGACCTGACTGACTACCTCA-3′; reverse: 5′-CAGCTTCTCCTTAATGTCACG-3′). Real-time quantitative PCR was performed in a 25 µl solution containing 100 ng of RNA templet, 0.3 µM of primers, and SYBR Green 12.5 µl. The value of threshold cycle (Ct) was generated at every cycle during a run. Fluorescent reading from real-time PCR reaction was quantitatively analyzed by determining the difference of Ct (delta Ct) between Ct of *TYR* gene and Ct of *ß-actin* gene. The gene expression of TYR was determined by the formation of 2^−delta Ct^ as we reported previously [43], [52]. The relative gene expression was expressed as percentage of non-UVB control.

### Statistical Analysis

All data were collected from three or more independent experiments and values were expressed as means ± SD. Statistical analysis was performed by one-way analysis of variance (ANOVA) using original software (Microcal Inc., Northampton, Mass., USA). p values lower than 0.05 were considered statistically significant difference.

## Results

### Doses of UVB Light as Determined by MTT Assay

**Figure 1:**
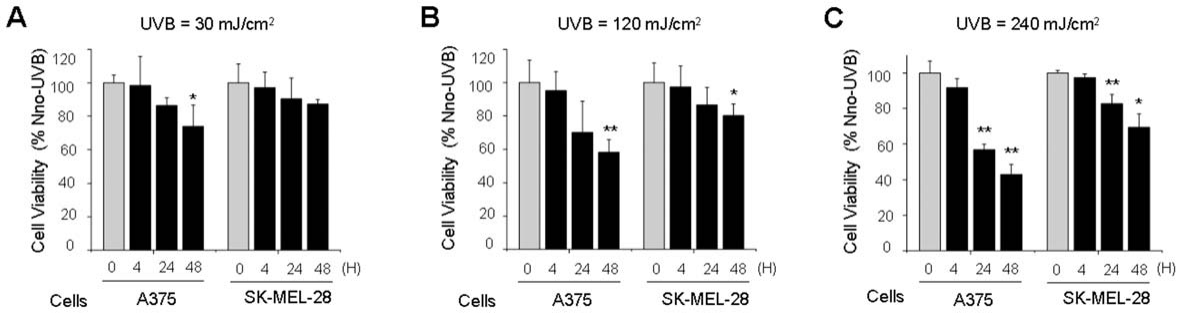
UVB light-induced loss of cell viability. A375 and SK-MEL-28 melanoma cells were exposed to UVB light at the doses of 30 mJ/cm^2^ (A), 120 mJ/cm^2^ (B) and 240 mJ/cm^2^(C) and post-cultured for 4 h, 24 h and 48 h (A, B, C). Cell viability was determined by MTT assay. The results were expressed as percentage of non-UVB control. *: p<0.05; **: p<0.01 as compared to its non-UVB control (0 h).

MTT assay showed that UVB light exposure caused loss of cell viability in both A375 and SK-MEL-28 melanoma cells time- and dose-dependently (Fig. 1A, 1B, 1C). At the dose of 120 mJ/cm^2^, the cell viability was significantly decreased by 42% in A375 and by 20% in SK-MEL-28 melanoma cells 48 h after UVB light exposure (Fig. 1B), whereas, the changes were not significant 4 and 24 h after UVB light exposure. Hence, a dose of UVB at 120 mJ/cm^2^ and post-culture for 24 h were chosen for further experiments.

### Differential Roles of α-Syn in Melanin Synthesis

**Figure 2:**
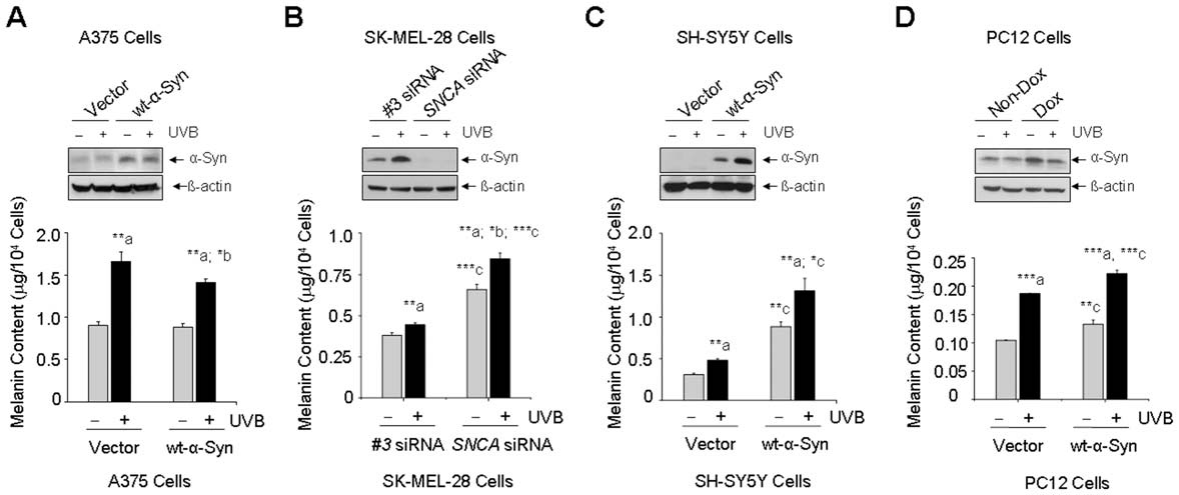
Roles of α-Syn in melanin synthesis in melanoma and dopaminergic neuronal cells. A375 melanoma cells (A) and dopaminergic neuronal SH-SY5Y and PC12 cells (C, D) with or without wt-α-Syn over-expression, SK-MEL-28 melanoma cells (B) with or without suppression of endogenous α-Syn, were exposed to UVB light (120 mJ/cm^2^) or non-UVB light and post-cultured for 24 h. The protein levels of α-Syn were determined by western blot assay (A, B, C, D, upper panel). Melanin content was determined spectrophotometrically at 415 nm using iMark Microplate Reader (A, B, C, D, lower panel). *: p<0.05; **: p<0.01; *** p<0.001 as compared to its non-UVB control (a), non-α-Syn control (b), or vector/*#3* siRNA control (c). Dox  =  doxycycline.

Western blot assay showed that transfection of cells with wt-α-Syn expression plasmid increased α-Syn protein level in both A375 melanoma cells (Fig. 2A, upper panel) and in SH-SY5Y dopaminergic neuronal cells (Fig. 2C, upper panel). It also showed that the protein level of α-Syn was higher in the inducible PC12 cells treated with doxycycline than that in non-doxycycline-treated PC12 cells (Fig. 2D, upper panel). Moreover, the endogenous expression of α-Syn was decreased in SK-MEL-28 melanoma cells transfected with *SNCA* siRNA for 72 h (Fig. 2B, upper panel), indicating the suppression of α-Syn.

In the parallel experiments, we found that UVB light irradiation increased melanin synthesis by 184%, 155% and by 179% in non-α-Syn-overexpressed A375 melanoma cells, SH-SY5Y and PC12 neuronal cells respectively, whereas, it was increased by 160%, 148% and by 167% in wt-α-Syn-over-expressed A375 melanoma cells, SH-SY5Y and PC12 neuronal cells as compared to its non-UVB light-exposed control cells (Fig. 2A, 2C, 2D, lower panel). Moreover, the melanin content was increased by 128% and by 117% respectively in SK-MEL-28 melanoma cells with or without suppression of endogenous α-Syn by *SNCA* siRNA transfection (Fig. 2B, lower panel).

Since UVB light-exposed neuronal cells do not represent cellular models of PD, we further analyzed roles of α-Syn in melanin content in PD cellular models, including α-Syn-expressed SH-SY5Y and PC12 dopaminergic neuronal cells. Different from melanoma cells, we found that, under the conditions of both non-UVB and UVB light exposure, the melanin content was 2.9 and 2.7 times higher in α-Syn-over-expressed SH-SY5Y cells than that in its non-α-Syn-expressed SH-SY5Y control cells (Fig. 2C, lower panel) and it was 1.3 and 1.2 times higher in α-Syn-expressed PC12 cells (treated with doxycycline) than that in its non-α-Syn-induced control PC12 cells (Fig. 2D, lower panel), indicating that α-Syn could be associated with an increase of melanin content in dopaminergic neuronal cells.

### Inhibitory Effects α-Syn on Tyrosinase (TYR) Activity

**Figure 3:**
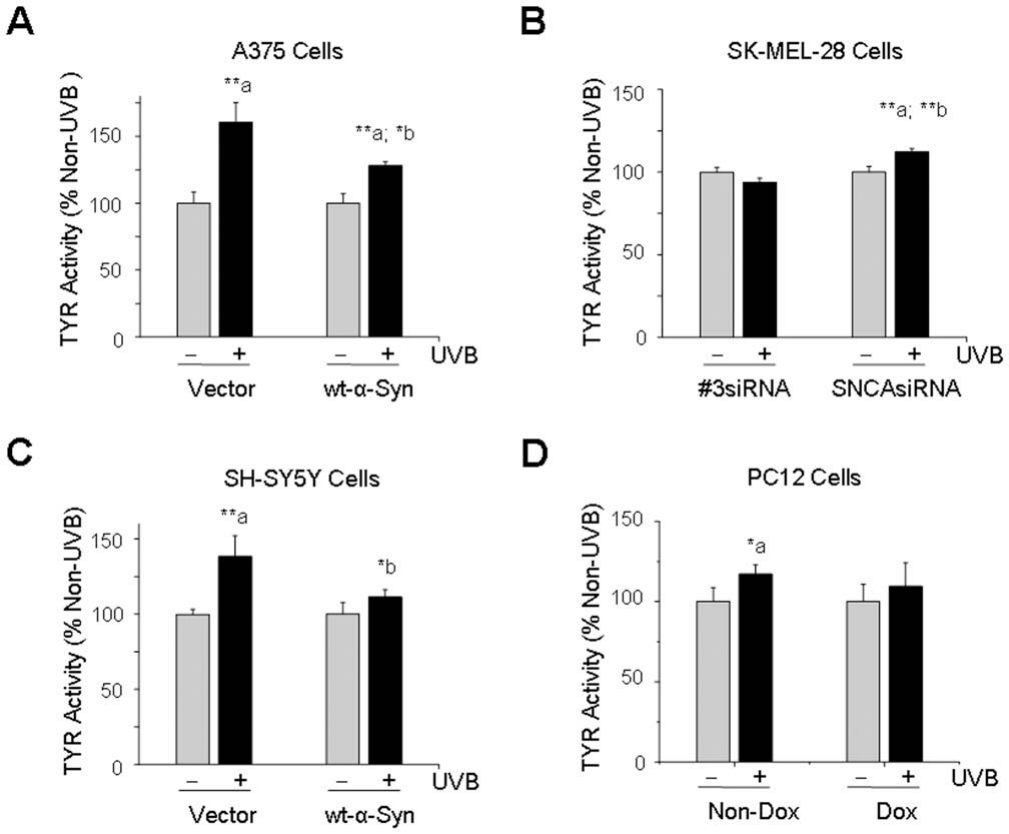
Effects of α-Syn on the activation of tyrosinase (TYR) induced by UVB light. After cells were exposed to UVB light (120 mJ/cm^2^) and post-cultured for 24 h, the TYR activity was measured spectrophotometrically at 475 nm using iMark Microplate Reader. Cells without UVB light exposure (non-UVB) were used as a control. A: A375 melanoma cells; B: SK-MEL-28 melanoma cells; C: SH-SY5Y dopaminergic neuronal cells; D: PC12 dopaminergic neuronal cells. *: p<0.05; **: p<0.01 as compared to its non-UVB control (a) or non-α-Syn control (b). Dox  =  doxycycline.

TYR is one of the critical enzymes for the initiation of melanogenesis [54], [55]. Our results showed that UVB light exposure was associated with an increase of TYR activity by 160%, 138% and by 117% in non-α-Syn over-expressed A375, SH-SY5Y and PC12 cells, whereas, it was increased by 127%, 111% and 109% in α-Syn over-expressed A375, SH-SY5Y and PC12 cells as compared to its non-UVB light-exposed control cells (Fig. 3A, 3C, 3D). Our results also revealed that UVB light exposure caused an increase of TYR activity by 112% in α-Syn-suppressed SK-MEL-28 melanoma cells as compared to its non-UVB light-exposed control cells (Fig. 3B). However, the change was not significant in SK-MEL28 melanoma cells transfected with *#3* siRNA, in which, endogenous α-Syn was expressed (Fig. 3B).

### α-Syn Expression Decreases Extra-cellular DA Content

**Figure 4:**
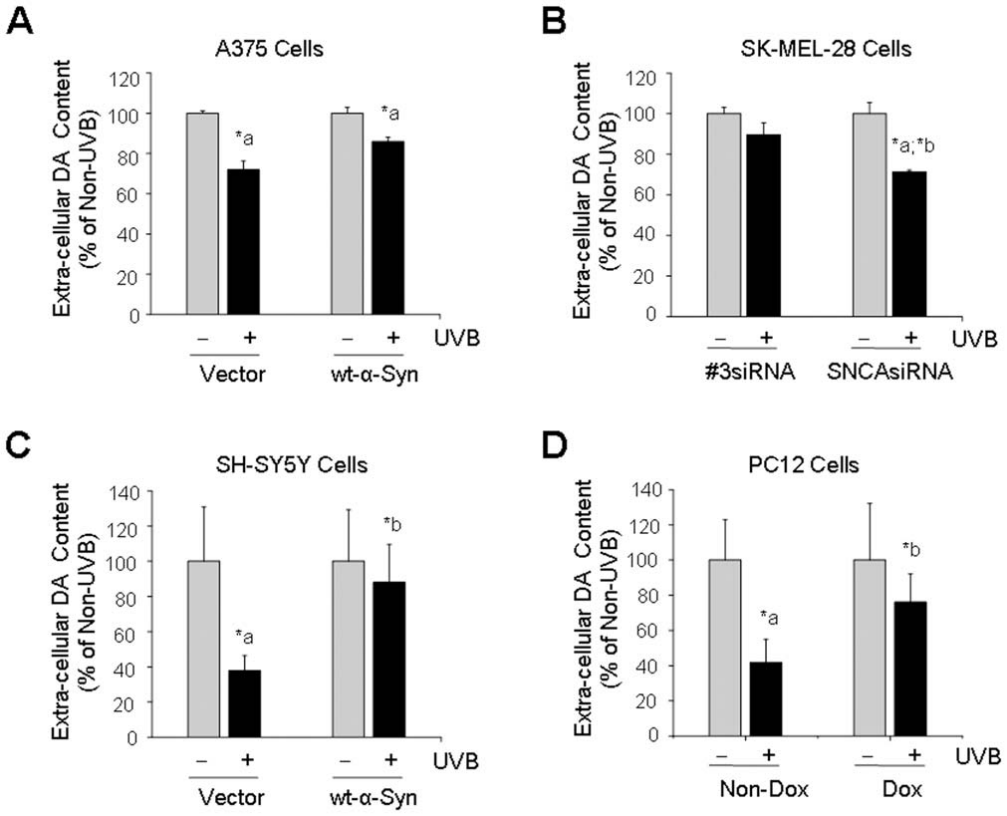
Extra-cellular dopamine (DA) content as determined by HPLC assay. After cells were exposed to UVB light (120 mJ/cm^2^) or non-UVB light and post-cultured for 24 h, cell culture medium from melanoma A375 cells (A) and SK-MEL-28 cells (B), dopaminergic neuronal SH-SY5Y cells (C) and PC12 cells (D) with or without α-Syn expression were subjected to HPLC assay for measuring DA level. *: p<0.05 as compared to its non-UVB control (a) or non-α-Syn control (b). Dox  =  doxycycline.

Both melanoma cells and dopaminergic neuronal cells share a common pathway in the initiation of biosynthesis of melanin and DA from tyrosine. In this study, we measured extra-cellular DA level from cell culture medium, which is an indicator of DA release. HPLC assay revealed that UVB light exposure caused decrease of extra-cellular DA level by 28%, 62% and 58% in non-α-Syn-expressed A375, SH-SY5Y and PC12 cells respectively, whereas, it was decreased by 14%, 12% and 24% in those cells over-expressed with α-Syn as compared to its non-UVB-exposed control cells (Fig. 4A, 4C, 4D). After endogenous α-Syn-expressed SK-MEL-28 melanoma cells were exposed to UVB light and post-cultured for 24 h, there was no significant change in the content of extra-cellular DA (Fig. 4B). However, when α-Syn was suppressed by *SNCA* siRNA transfection, the extra-cellular DA level was decreased by 29% as compared to it non-UVB-exposed control cells (Fig. 4B).

### Role of α-Syn in the Expression of TYR

**Figure 5:**
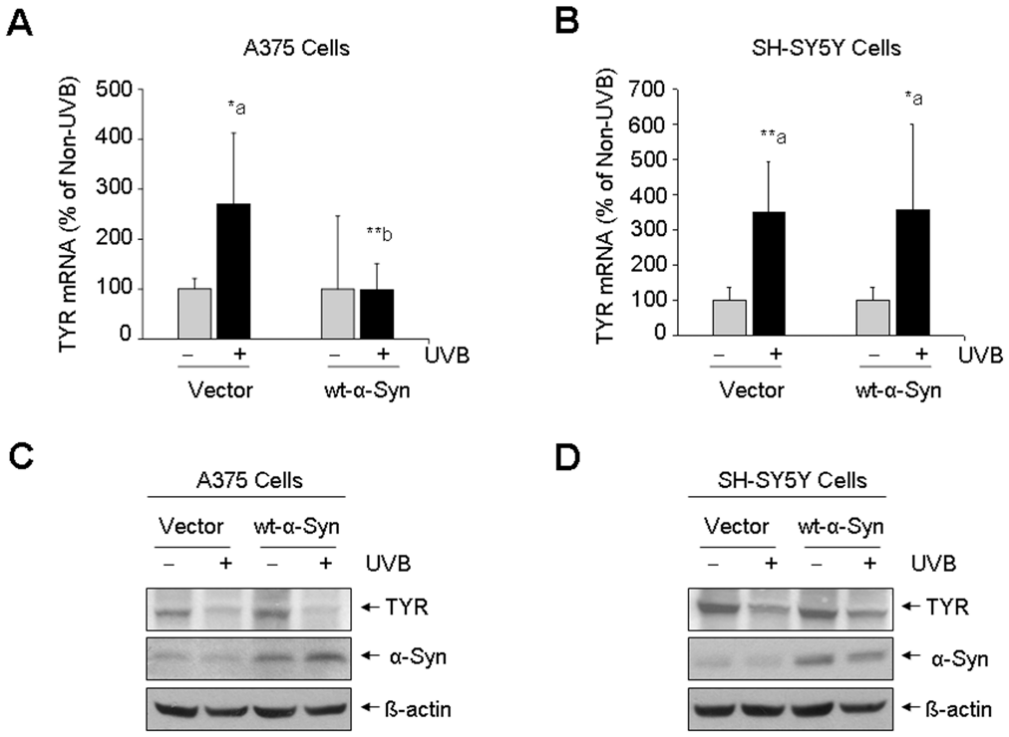
UVB light-induced changes in gene expression and protein level of TYR. A375 (A) and SH-SY5Y cells (B) with or without α-Syn expression were exposed to UVB light (120 mJ/cm^2^) or non-UVB light and post-cultured for 24 h. Gene expression of TYR was determined by real-time quantitative PCR assay (A, B). *: p<0.05; **: p<0.01 as compared to its non-UVB control (a) or non-α-Syn control (b). The protein levels of TYR were determined by western blot assay (C, D).

We further evaluated whether the changes in TYR activity were related to the changes of TYR expression. Real-time quantitative PCR revealed that UVB light exposure significantly increased TYR mRNA level by 270% in non-α-Syn-expressed A375 melanoma cells, whereas, there was no significant change in α-Syn-over-expressed A375 melanoma cells (Fig. 5A). We also showed that UVB light exposure caused a significant increase of TYR gene expression by 357% in non-α-Syn-expressed SH-SY5Y cells and by 349% in α-Syn-expressed SH-SY5Y cells (Fig. 5B). However, western blot assay did not show an increase of TYR protein level in either A375 melanoma cells (Fig. 5C) or in SH-SY5Y dopaminergic neuronal cells (Fig. 5D).

## Discussion

UVR–induced DNA damage is considered as one of the key pathogenic mechanisms underlying the development of melanoma [12], and UVR-induced melanin synthesis is a major physiological defense against solar irradiation-induced DNA damage by preventing the penetration of UVR and by absorbing or scattering the UVR [14], [15], [49]. The induction of melanin synthesis, stimulation of TYR activity, and triggering the MSH/melanocortin 1 receptor (MC1R)/cAMP pathway are critical steps in UVR-induced melanogenesis [56]–[58]. Consistent with previous reports, we showed that UVB light exposure increased melanin synthesis as well as TYR activity. Our results that α-Syn expression reduced UVB light-induced increase of melanin synthesis and that the melanin content was less when melanoma cells were expressed with α-Syn indicate that α-Syn may have inhibitory effects on UVB light-induced melanin synthesis in melanoma cells, which were supported by the recent findings that α-Syn-expressed melanoma cells generate no or a very low level of melanin pigments [31].

**Figure 6:**
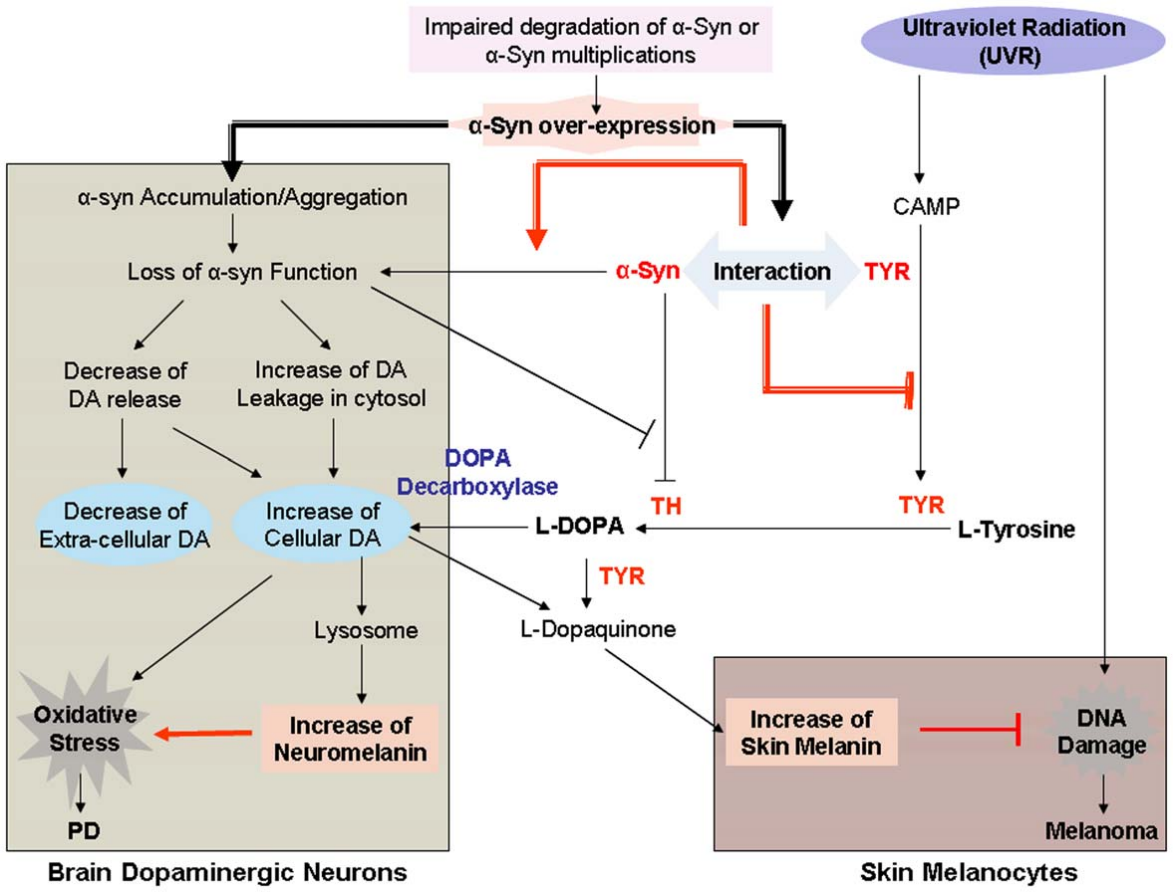
Flow chat of the linkage between PD and melanoma mediated by α-Syn. In skin melanocytes, UVR causes DNA damage, leading to the initiation of melanoma. UVR also induces melanin synthesis which is catalyzed by TYR. Increased melanin prevents UVR-induced DNA damage, reducing the vulnerability to development of melanoma. α-Syn expression in skin melanocytes may interact with TYR, inhibiting UVR-induced TYR activation, leading to the reduction of melanin synthesis, which may enhance the susceptibility of skin melanocytes to develop melanoma. In brain dopaminergic neurons, impaired degradation of α-Syn or α-Syn multiplications causes accumulation/aggregation of α-Syn, leading to the loss of α-Syn function, thereafter, the decrease of DA release and increase of cellular DA content, which may be converted into melanin in lysosome. The increased melanin content in dopaminergic neurons (neuromelanin) enhances the susceptibility to oxidative stress-induced neuronal injury relevant to PD.

Melanoma cells are malignant tumor cells of melanocytes, expressing mainly the rate-limiting enzyme TYR involved in the biosynthesis of melanin [34]. Consistent with the results from melanin assay, α-Syn over-expression demonstrated a less increase of TYR activity as compared to that in non-α-Syn-expressed control cells, indicating that α-Syn may also inhibit UVB light-induced activation of TYR enzyme. The interaction between α-Syn and TYR could be the explanation for the reduction of UVB light-induced increase of melanin synthesis and TYR activity in α-Syn-expressed melanoma cells (Fig. 6).

Different from melanoma cells, dopaminergic neuronal cells express mainly TH enzyme involved in the biosynthesis of DA, and accordingly, neuromelanin (NM), melanin presented in dopaminergic neurons. It has been reported that accumulation of α-Syn can result in impaired SNAE-complex assembly and synaptic vesicle function [59] and the loss of TH inhibition, which in turn, can reduce the release of DA and increase the content of cytosolic DA, thereafter, the increase of melanin level (Fig. 6). We explain that UVB light-induced decrease of extra-cellular DA content in dopaminergic neuronal cells could be related to the increased synthesis of neuromelanin from cellular DA in lysosome.

Since NM is produced from oxyradical metabolites of monoamine neuroamine neurotransmitters including DA [60], the observed increase of melanin content in α-Syn over-expressed dopaminergic neuronal SH-SY5Y and PC12 cells, cellular models relevant to PD, could be mainly explained by the increased cytosolic DA due to the loss of α-Syn function on the inhibition of TH. Our experimental result is also supported by the recent report that increased expression of α-Syn is associated with NM accumulation [61].

Although NM can interact with free iron in SN neurons, acting as a strong neuron protectant and a key factor in delaying neuron death [62]–[65], the interaction of NM with iron can also make dopaminergic nigral neurons more sensitive to oxidative stress, leading to neuronal injury [66]–[69]. Therefore, the increase of melanin in dopaminergic neurons correlates with enhanced susceptibility to oxidative stress-induced neuronal injury relevant to PD (Fig. 6). The reports that NM enhances the toxicity of α-Syn in SK-N-SH cells [70] and that the selective neurotoxicity of α-Syn is dependent on DA [71] further support that the neurotoxicity of α-Syn could be related to the increased melanin content.

Although our results show that UVB light exposure increases TYR gene expression, which is consistent with previous reports, we did not show an increase of TYR protein level in UVB light-exposed cells. Previous reports have suggested that TYR mRNA, melanin and protein levels do not always correlate because of multiple factors, such as proteolytic degradation system and other auto-regulatory mechanisms [72]. This may provide an explanation for the seemingly inconsistent results between the changes of TYR mRNA and TYR protein levels in our study.

In conclusion, α-Syn over-expression causes less increase of UVB light-induced melanin synthesis in melanoma cells and causes higher melanin levels in dopaminergic neuronal cells. These differential roles of α-Syn in the biosynthesis of melanin in melanocytes and dopaminergic neurons may be critical in the association of these two diseases. Further studies are necessary to further evaluate roles of α-Syn in UVB irradiation-induced tumorigenesis of melanoma in normal skin melanocytes and to explore possible mechanisms involved. Melanoma is the most dangerous form of skin cancers with high metastasis rate and poor prognosis. Understanding the linkage between PD and melanoma by α-Syn may draw an attention to our physicians and patients that a periodic dermatological screening for neoplastic or pre-neoplastic skin lesions as well as for skin α-Syn expression by skin biopsy in PD patients is important as to diagnose early and prevent the development of melanoma.
